# Mechanotherapy: how physical therapists’ prescription of exercise promotes tissue repair

**DOI:** 10.1136/bjsm.2008.054239

**Published:** 2009-02-24

**Authors:** K M Khan, A Scott

**Affiliations:** University of British Columbia, Vancouver, Canada

## Abstract

Mechanotransduction is the physiological process where cells sense and respond to mechanical loads. This paper reclaims the term “mechanotherapy” and presents the current scientific knowledge underpinning how load may be used therapeutically to stimulate tissue repair and remodelling in tendon, muscle, cartilage and bone.

The purpose of this short article is to answer a frequently asked question “How precisely does exercise promote tissue healing?” This is a fundamental question for clinicians who prescribe exercise for tendinopathies, muscle tears, non-inflammatory arthropathies and even controlled loading after fractures. High-quality randomised controlled trials and systematic reviews show that various forms of exercise or movement prescription benefit patients with a wide range of musculoskeletal problems.^1^^–^4 But what happens at the tissue level to promote repair and remodelling of tendon, muscle, articular cartilage and bone?

The one-word answer is “mechanotransduction”, but rather than finishing there and limiting this paper to 95 words, we provide a short illustrated introduction to this remarkable, ubiquitous, non-neural, physiological process. We also re-introduce the term “mechanotherapy” to distinguish therapeutics (exercise prescription specifically to treat injuries) from the homeostatic role of mechanotransduction. Strictly speaking, mechanotransduction maintains normal musculoskeletal structures in the absence of injury. After first outlining the process of mechanotransduction, we provide well-known clinical therapeutic examples of mechanotherapy–turning movement into tissue healing.

## WHAT IS MECHANOTRANSDUCTION?

Mechanotransduction refers to the process by which the body converts mechanical loading into cellular responses. These cellular responses, in turn, promote structural change. A classic example of mechanotransduction in action is bone adapting to load. A small, relatively weak bone can become larger and stronger in response to the appropriate load through the process of mechanotransduction.^5^

We searched PUBMED, EMBASE, MEDLINE, CINAHL, Google, Wikipedia, Melways and various library collections for the earliest reference to “mechanotransduction”. The first paper referenced under this term is by McElhaney *et al* in volume 1 of the *Journal of Biomechanics*, but the term is not used in that paper.^6^ Although there are 2441 citations in MEDLINE for mechanotransduction, the word is not found in the current edition of the Oxford English Dictionary. A useful formal definition of mechanotransduction might be “the processes whereby cells convert physiological mechanical stimuli into biochemical responses”. Mechanotransduction is generally broken down into three steps: (1) mechanocoupling, (2) cell–cell communication and (3) the effector response. To simplify this for patients, these same elements can be thought of as (1) the mechanical trigger or catalyst, (2) the communication throughout a tissue to distribute the loading message and (3) the response at the cellular level to effect the response—that is, the tissue “factory” that produces and assembles the necessary materials in the correct alignment. The communication at each stage occurs via cell signalling—an information network of messenger proteins, ion channels and lipids. In the following section, we detail these three steps using the tendon as an illustration; the fundamental processes also apply to other musculoskeletal tissues.

### 1. Mechanocoupling

**Figure 1:**
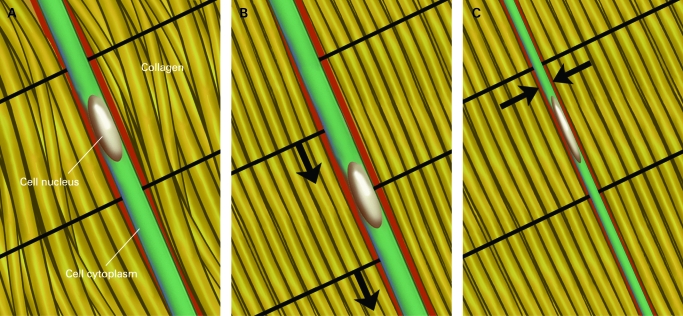
Tendon cell undergoing (A,B) shear and (C) compression during a tendon-loading cycle.

Mechanocoupling refers to physical load (often shear or compression) causing a physical perturbation to cells that make up a tissue. For example, with every step the Achilles tendon receives tensile loads generated by three elements of the gastrocnemius–soleus complex and thus, the cells that make up the tendon experience tensile and shearing forces. Tendons can also experience compression forces (fig 1A,B) These forces elicit a deformation of the cell that can trigger a wide array of responses depending on the type, magnitude and duration of loading.^7^ The key to mechanocoupling, as the name suggests, is the direct or indirect physical perturbation of the cell, which is transformed into a variety of chemical signals both within and among cells.

### 2. Cell–cell communication

**Figure 2:**
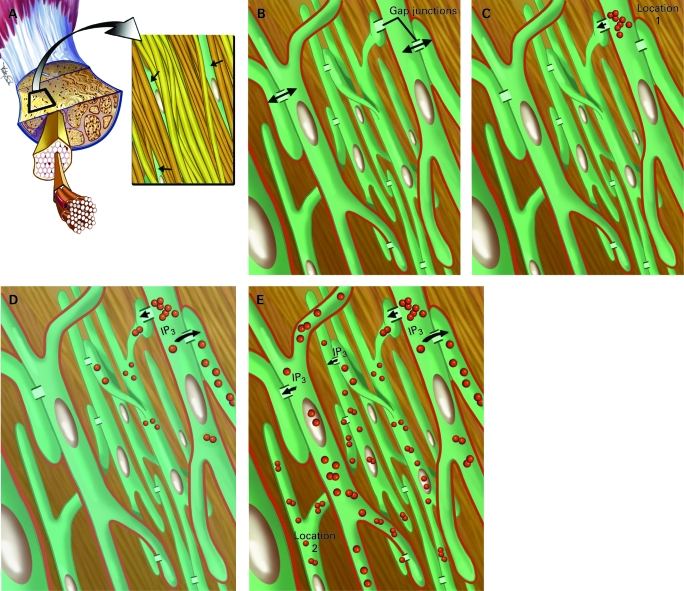
Tendon tissue provides an example of cell–cell communication. (A) The intact tendon consists of extracellular matrix (including collagen) and specialised tendon cells (arrowheads). (B) Tendon with collagen removed to reveal the interconnecting cell network. Cells are physically in contact throughout the tendon, facilitating cell–cell communication. Gap junctions are the specialised regions where cells connect and communicate small charged particles. They can be identified by their specific protein connexin 43. (C–E) Time course of cell–cell communication from (C) beginning, through (D) the midpoint to (E) the end. The signalling proteins for this step include calcium (red spheres) and inositol triphosphate (IP3).

The previous paragraph illustrated mechanocoupling by focusing on a single cell, but let us draw back to examine a larger tissue area that contains thousands of cells embedded within an extracellular matrix (fig 2). The signalling proteins for this step include calcium and inositol triphosphate. The process of cell–cell communication is best understood by illustration (fig 2) and animation (supplementary slides online). The critical point is that stimulus in one location (location “1” in fig 2C) leads to a distant cell registering a new signal (location “2” in fig 2E) even though the distant cell does not receive a mechanical stimulus.^7^

### 3. Effector cell response

**Figure 3:**
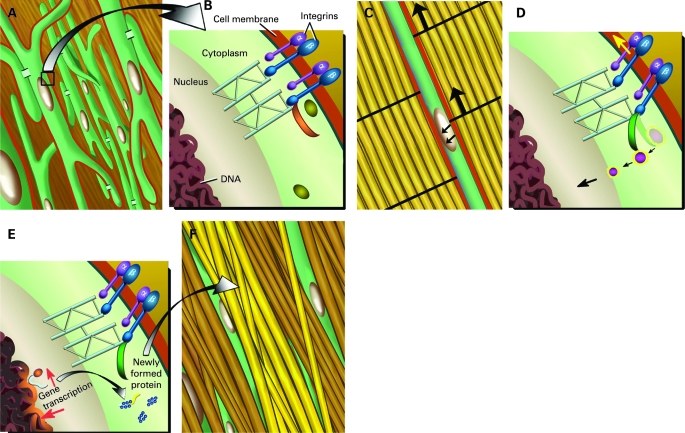
Mechanical loading stimulates protein synthesis at the cellular level. (A) A larger scale image of the tendon cell network for orientation. We focus on one very small region. (B) Zooming in on this region reveals the cell membrane, the integrin proteins that bridge the intracellular and extra-cellular regions, and the cytoskeleton, which functions to maintain cell integrity and distribute mechanical load. The cell nucleus and the DNA are also illustrated. (C) With movement (shearing is illustrated), the integrin proteins activate at least two distinct pathways. (D) One involves the cytoskeleton that is in direct physical communication with the nucleus (ie, tugging the cytoskeleton sends a physical signal to the cell nucleus). Another pathway is triggered by integrins activating a series of biochemical signalling agents which are illustrated schematically. After a series of intermediate steps those biochemical signals also influence gene expression in the nucleus. (E). Once the cell nucleus receives the appropriate signals, normal cellular processes are engaged. mRNA is transcribed and shuttled to the endoplasmic reticulum in the cell cytoplasm, where it is translated into protein. The protein is secreted and incorporated into extracellular matrix. (F) In sum, the mechanical stimulus on the outside of the cell promotes intracellular processes leading to matrix remodelling.

To illustrate the third part of mechanotransduction (effector cell response), we focus on the boundary between the extracellular matrix and a single cell (fig 3). This process can be harnessed by mechanotherapy to promote tissue repair and remodelling. The main steps in mechanotransduction for connective tissues have been essentially unravelled for bone, but there remain unknown elements in the load-induced signalling pathways for muscle,^8^ ^9^ tendon^10^^–^12 and articular cartilage.^13^ The reader seeking more detailed explanations of the process of protein synthesis generally is referred to classic texts (eg, Alberts *et al*14]). For more detailed explanations of mechanotransduction in connective tissue please consider the work of Ingber,^15^^–^18 Arnoczky,^10^ ^19^ ^20^ Banes,^21^^–^28 and Hart.^29^^–^32

To briefly summarise, it seems that mechanotransduction is an ongoing physiological process in the human body, just like respiration and circulation. Consider the skeleton as an example of a connective tissue; the body’s sensor is the osteocyte network and the process of regulating bone to load has been referred to as the “mechanostat”.^33^ ^34^ In the absence of activity, the mechanotransduction signal is weak, so connective tissue is lost (eg, osteoporosis). When there are loads above the tissue’s set point, there is a stimulus through mechanotransduction so that the body adapts by increasing protein synthesis and adding tissue where possible (larger, stronger bone).^33^ ^34^

## MECHANOTHERAPY: THE CLINICAL APPLICATION OF MECHANOTRANSDUCTION

To test whether this important process was being taught in physical therapy curricula we formed international, intergenerational focus groups. Our informal “results” (unpublished data) suggested that mechanotransduction was not being taught as an important biological principle in physical therapy programmes. The same applied to medicine but we did not expect medical education to include this topic as most medical schools only allocate a perfunctory hour to the fact that physical activity is medicine.^35^ This is a major failing of medical education when physical inactivity is the major public health problem of the 21st century.^36^

To highlight the crucial role of mechanotransduction in underpinning musculoskeletal rehabilitation, we propose to re-introduce the term “mechanotherapy” for those many situations where therapeutic exercise is prescribed to promote the repair or remodelling of injured tissue. Mechanotherapy was first defined in 1890 as “the employment of mechanical means for the cure of disease” (Oxford English Dictionary). We would update this to “the employment of mechanotransduction for the stimulation of tissue repair and remodelling.” This distinction highlights the cellular basis of exercise prescription for tissue healing and also recognises that injured and healthy tissues may respond differently to mechanical load. Databases and library searches did not reveal the term mechanotherapy being used in other ways in physical therapy.

To close off this introductory piece we summarise clinical studies that have shown or implied a potential for mechanotherapy to promote healing of tendon, muscle, cartilage and bone.

## SUMMARY OF CLINICAL STUDIES

### Tendon

Tendon is a dynamic, mechanoresponsive tissue. One of the major load-induced responses shown both in vitro^24^ and in vivo^31^ ^37^ ^38^ in tendon is an upregulation of insulin-like growth factor (IGF-I). This upregulation of IGF-I is associated with cellular proliferation and matrix remodelling within the tendon. However, recent studies suggest that other growth factors and cytokines in addition to IGF-I are also likely to play a role.^39^ Alfredson *et al* examined tendon structure by grey-scale ultrasound in 26 tendons with Achilles tendinosis, which had been treated with eccentric exercise. Remarkably, after a mean follow up of 3.8 years, 19 of 26 tendons had a more normalised structure, as gauged by their thickness and by the reduction of hypoechoic areas.^40^ This study and others^41^ show that tendon can respond favourably to controlled loading after injury. Research into the ideal loading conditions for different types of tendon injury is still ongoing.

### Muscle

Muscle offers one of the best opportunities to exploit and study the effects of mechanotherapy, as it is highly responsive to changes in functional demands through the modulation of load-induced pathways. Overload leads to the immediate, local upregulation of mechanogrowth factor (MGF), a splice variant of IGF-I with unique actions.^42^ MGF expression in turn leads to muscle hypertrophy via activation of satellite cells.^42^ The clinical application of mechanotherapy for muscle injury is based on animal studies.^43^ After a brief rest period to allow the scar tissue to stabilise, controlled loading is started. The benefits of loading include improved alignment of regenerating myotubes, faster and more complete regeneration, and minimisation of atrophy of surrounding myotubes.^43^

### Articular cartilage

Like other musculoskeletal tissues, articular cartilage is populated by mechanosensitive cells (chondrocytes), which signal via highly analogous pathways. Alfredson and Lorentzon treated 57 consecutive patients with isolated full-thickness cartilage defect of the patella and disabling knee pain of long duration by periosteal transplantation either with or without continuous passive motion (CPM). In this study, 76% of patients using CPM achieved an “excellent” outcome, whereas only 53% achieved this in the absence of CPM.^44^ Tissue repair was not directly assessed in this case series, but the results encourage further research into the underlying tissue response and the optimisation of loading parameters.

### Bone

In bone, osteocytes are the primary mechanosensors. A recent clinical study suggested that the beneficial effect of mechanotransduction may be exploited by appropriately trained physical therapists to improve fracture healing. In this study, 21 patients with a distal radius fracture were randomised to receive (1) standard care including immobilisation and gripping exercises or (2) standard care plus intermittent compression delivered via an inflatable pneumatic cuff worn under the cast. The experimental group displayed significantly increased strength (12–26%) and range of motion (8–14%) at the end of the immobilisation period and these differences were maintained at 10 weeks.^45^^–^47 Future, larger studies are planned by this group to confirm whether the effects of compression affected the fracture healing itself, as suggested by preclinical studies with similar loading parameters.^45^^–^47
